# Supplementary material for: Complexome profiling of the Chlamydomonas psb28 mutant reveals TEF5 as an early PSII assembly factor
Source: Plant Cell. 2025 Mar 17;37(6):koaf055. doi: 10.1093/plcell/koaf055 (PMC12164590; doi:10.1093/plcell/koaf055)
Supplement: koaf055_Supplementary_Data [file koaf055_supplementary_data.zip › psb28_interactive.zip!/plots/intensity/qualityPlots/peptides/atpE_Cre-1.g2716972.t1.p1.html]

### Cre-1.g2716972.t1.p1

Trivial name: atpE  
SeqCoverage [median]: 77.30  
Mass: 15.37 kDa  
  
 

| Sequence | MissedCleavage | Mass [Da] | wtIntensity | muIntensity | Score |
| --- | --- | --- | --- | --- | --- |
| AEGVKEKVEANFAYK | 2 | 1681.87 | 0.00 | 2932.60 | 74.77 |
| AEGVKEKVEANFAYKR | 3 | 1837.97 | 1038.61 | 2980.39 | 162.29 |
| ANLEKAEGVK | 1 | 1057.58 | 9547.12 | 16707.59 | 141.71 |
| ANLEKAEGVKEK | 2 | 1314.71 | 10474.28 | 10710.48 | 138.42 |
| DAFETAK | 0 | 780.37 | 71461.61 | 55953.23 | 143.56 |
| EKVEANFAYK | 1 | 1197.60 | 140525.50 | 82667.00 | 149.27 |
| EKVEANFAYKR | 2 | 1353.70 | 22727.78 | 38444.23 | 179.94 |
| GGQASGSKDEWNSYAIMGGFALVK | 1 | 2472.17 | 73935.67 | 64469.41 | 156.41 |
| QNQVTILANEAVSAENINPEEAK | 0 | 2481.23 | 29818.28 | 31256.33 | 195.09 |
| QNQVTILANEAVSAENINPEEAKDAFETAK | 1 | 3243.59 | 77154.67 | 101999.58 | 156.58 |
| SLQISILTPERPFWNGQADEIILPTETGEMGVLK | 1 | 3781.96 | 2438.21 | 0.00 | 43.24 |
| VEANFAYK | 0 | 940.47 | 255655.45 | 156422.27 | 148.38 |
| VEANFAYKR | 1 | 1096.57 | 105914.14 | 126056.18 | 118.71 |
|  |  |  | 800691.3 | 690599.3 |  |
